# Supplementary material for: In Silico Comparison Shows that the Pan-Genome of a Dairy-Related Bacterial Culture Collection Covers Most Reactions Annotated to Human Microbiomes
Source: Microorganisms. 2020 Jun 27;8(7):966. doi: 10.3390/microorganisms8070966 (PMC7409220; doi:10.3390/microorganisms8070966)
Supplement: Supplementary file 1 [file microorganisms-08-00966-s001.zip › Supplementary_Figure_S1.docx]

**
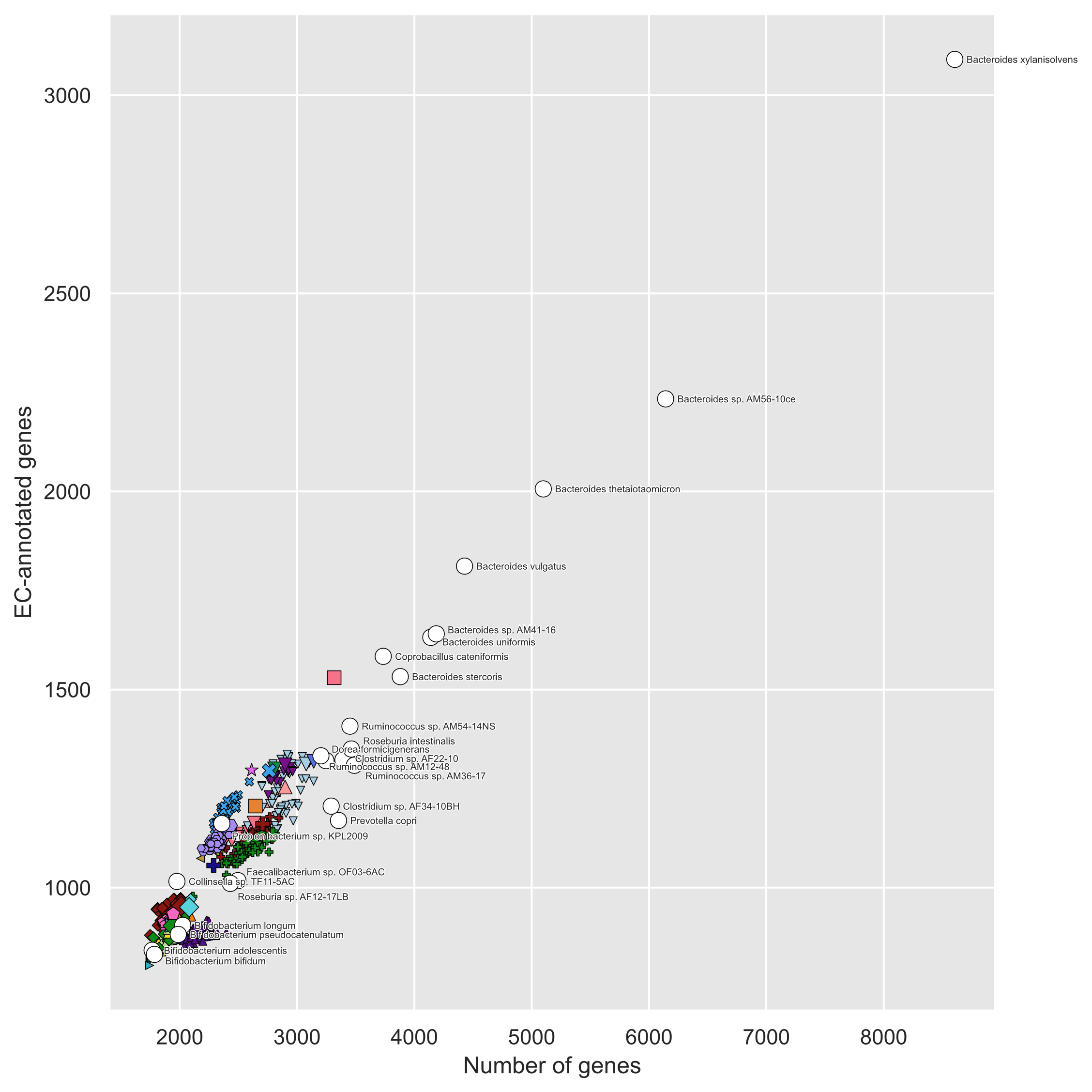
**

**Figure S1.** Relationship between the number of identified genes and the number of EC-annotated genes for the Liebefeld collection strains (colored symbols, each species is represented by a unique symbol and color) and 24 human gut bacteria randomly selected from Zou et al. [43] (white dots, the strain description is written next to the dot). Strains from the Liebefeld selection are highlighted with a larger symbol.
