# Supplementary material for: In Silico Comparison Shows that the Pan-Genome of a Dairy-Related Bacterial Culture Collection Covers Most Reactions Annotated to Human Microbiomes
Source: Microorganisms. 2020 Jun 27;8(7):966. doi: 10.3390/microorganisms8070966 (PMC7409220; doi:10.3390/microorganisms8070966)
Supplement: Supplementary file 1 [file microorganisms-08-00966-s001.zip › Supplementary_Figure_S2.docx]

**
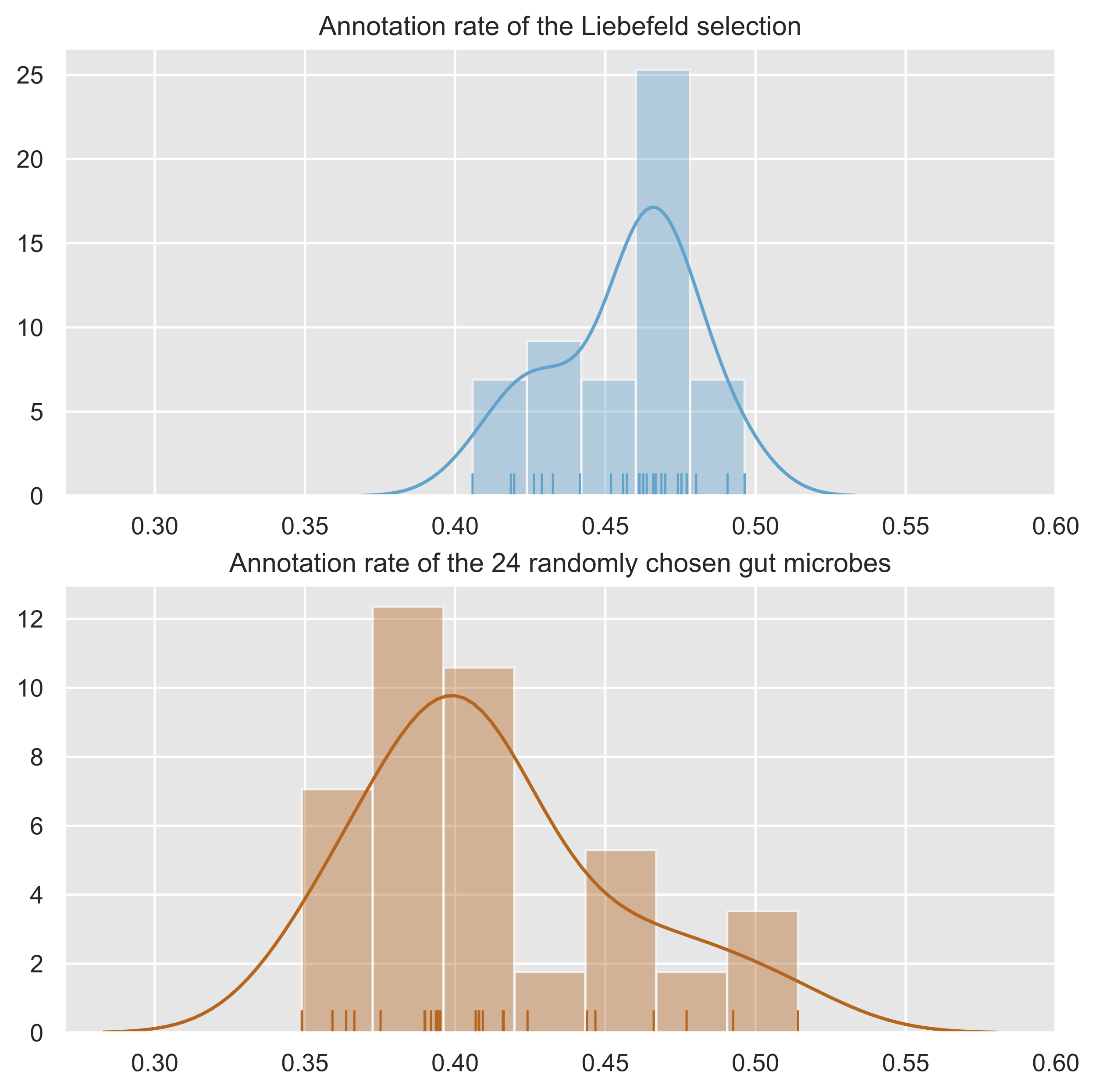
**

**Figure S2.** Histogram of the annotation rates for the Liebefeld selection strains (top, blue) and the 24 human gut bacteria randomly selected from Zou et al. [43] (bottom, brown). The distributions of the annotation rate are significantly different (Mann–Whitney U test, *p*-value = 1.68 × 10^-4^).
