# Supplementary material for: In Silico Comparison Shows that the Pan-Genome of a Dairy-Related Bacterial Culture Collection Covers Most Reactions Annotated to Human Microbiomes
Source: Microorganisms. 2020 Jun 27;8(7):966. doi: 10.3390/microorganisms8070966 (PMC7409220; doi:10.3390/microorganisms8070966)
Supplement: Supplementary file 1 [file microorganisms-08-00966-s001.zip › Supplementary_Figure_S3.docx]

**
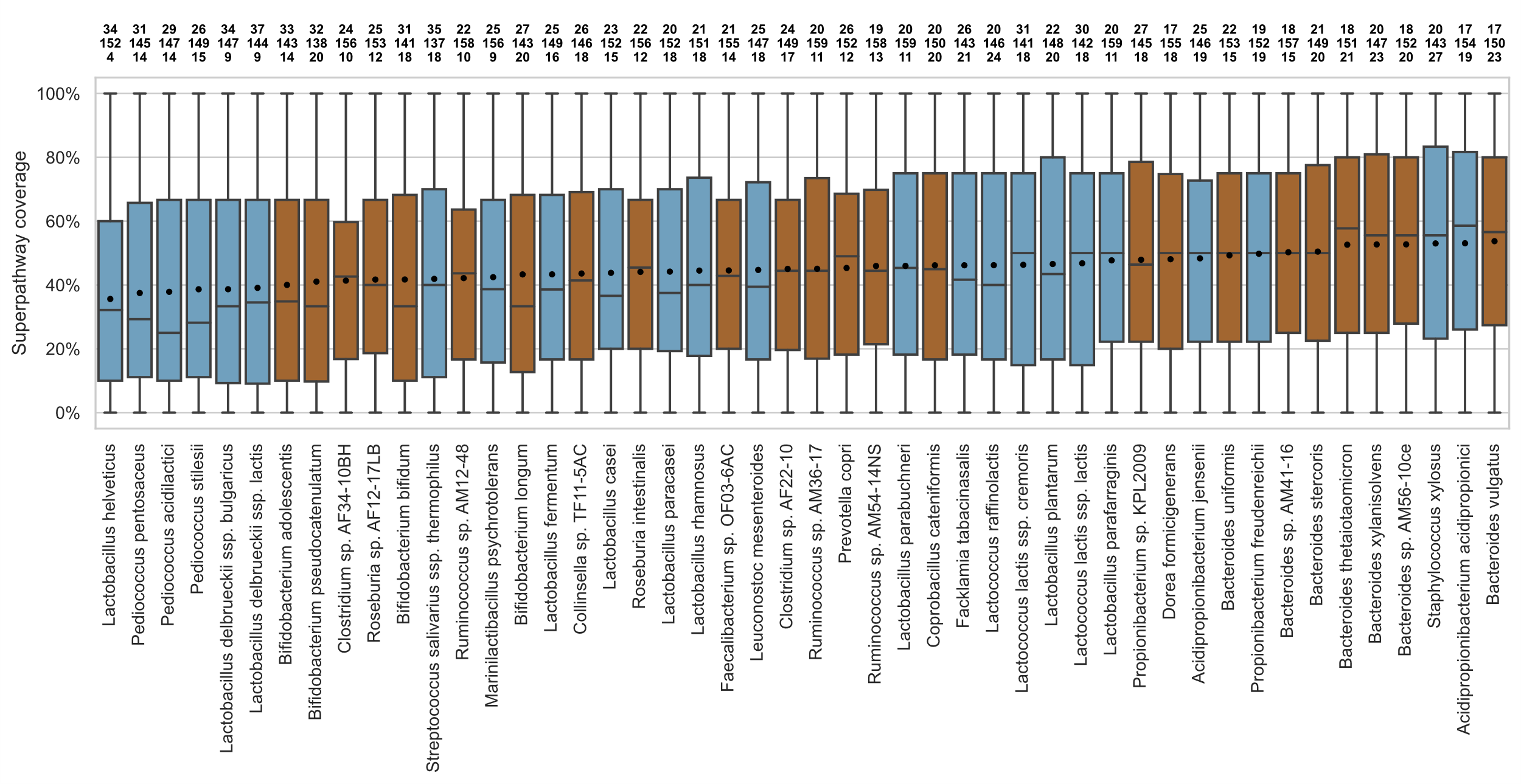
**

**Figure S3.** Boxplot of the coverage of the 190 MetaCyc superpathways by each of the 24 strains of the Liebefeld selection (blue, referred to by their species name) and 24 human gut bacteria randomly selected from Zou et al. [43] (brown, referred to by their NCBI organism name). The strains or sets of strains are sorted in ascending order according to their mean superpathway coverage, indicated by a black dot. The difference in the distribution of the mean superpathway coverage is not significant (Mann–Whitney U test, *p*-value = 0.32). Above each boxplot, three numbers indicate how many superpathways are not covered (top row), partially covered (middle row) and completely covered (bottom row).
