# Supplementary material for: In Silico Comparison Shows that the Pan-Genome of a Dairy-Related Bacterial Culture Collection Covers Most Reactions Annotated to Human Microbiomes
Source: Microorganisms. 2020 Jun 27;8(7):966. doi: 10.3390/microorganisms8070966 (PMC7409220; doi:10.3390/microorganisms8070966)
Supplement: Supplementary file 1 [file microorganisms-08-00966-s001.zip › Supplementary_Figure_S4.docx]

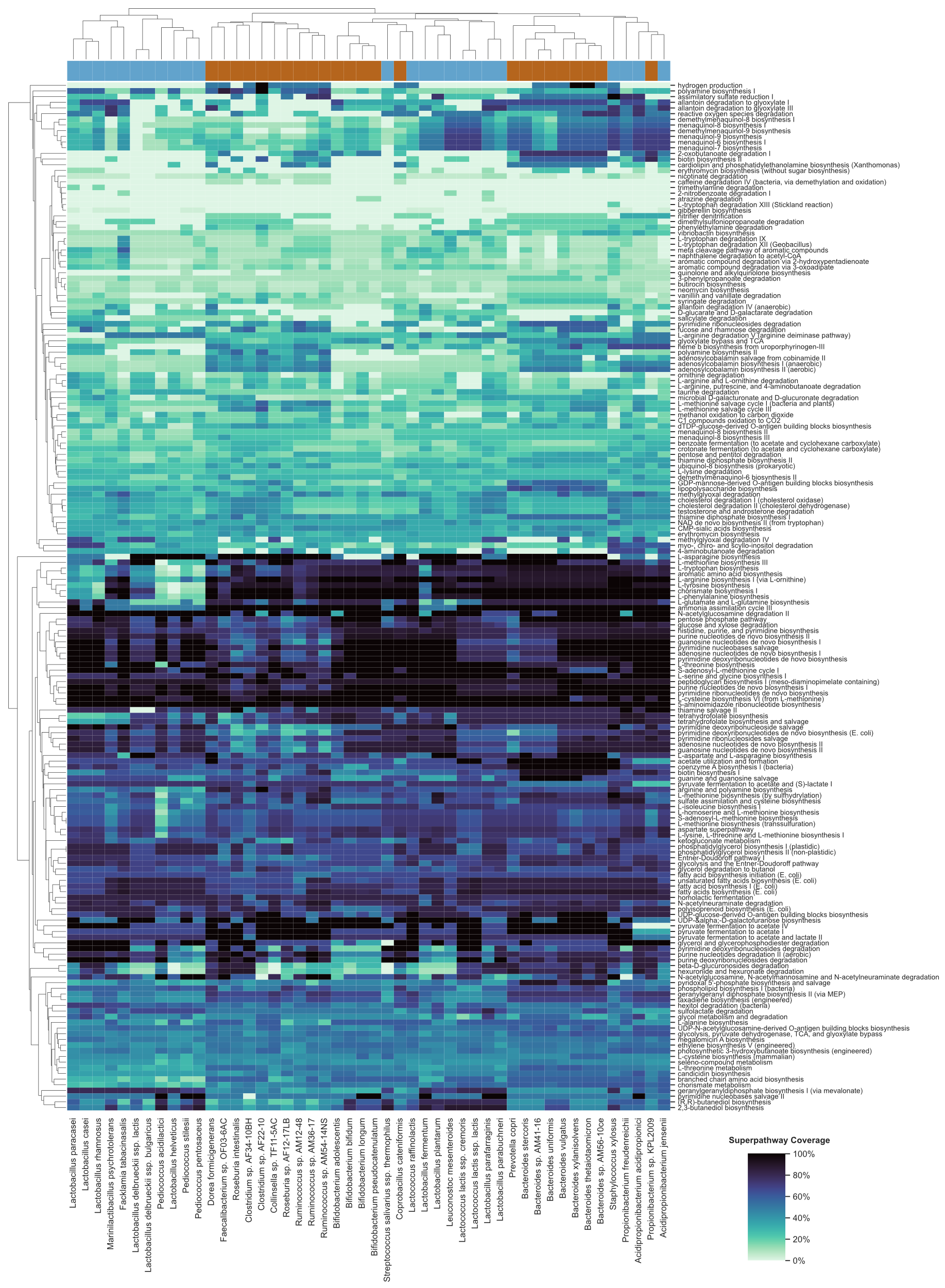


**Figure S4.** Overview of the biochemical potential of the 24 strains of Liebefeld selection (blue, referred to by their species name) and 24 human gut bacteria randomly selected from Zou et al. [43] (brown, referred to by their NCBI organism name). The Y-axis denotes the 190 superpathways of MetaCyc. The dendrogram of both axes resulted from hierarchical clustering. The colors of the heatmap denote superpathway coverage and range from white (0%) to black (100%).
