# Supplementary material for: In Silico Comparison Shows that the Pan-Genome of a Dairy-Related Bacterial Culture Collection Covers Most Reactions Annotated to Human Microbiomes
Source: Microorganisms. 2020 Jun 27;8(7):966. doi: 10.3390/microorganisms8070966 (PMC7409220; doi:10.3390/microorganisms8070966)
Supplement: Supplementary file 1 [file microorganisms-08-00966-s001.zip › Supplementary_Figure_S5.docx]

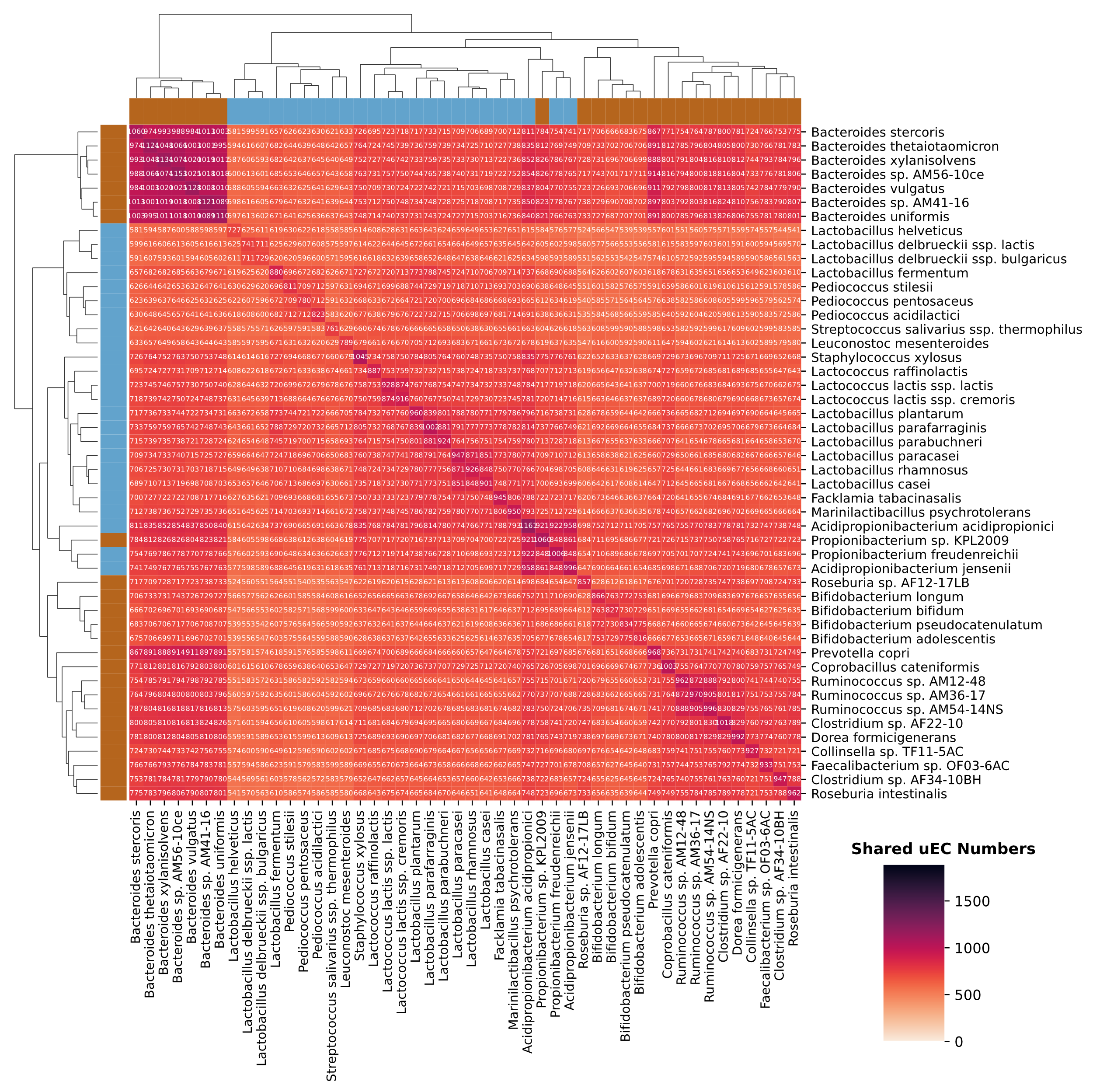


**Figure S5.** Clustered heatmap of the number of shared unique EC numbers (uECs) of 24 strains of Liebefeld selection (blue, referred to by their species name) and 24 human gut bacteria randomly selected from Zou et al. [43] (brown, referred to by their NCBI organism name). The total number of uECs annotated to a species/metagenome can be read from the antidiagonal line, where the same species intersect. The dendrogram of both axes resulted from hierarchical clustering.
