# Supplementary material for: In Silico Comparison Shows that the Pan-Genome of a Dairy-Related Bacterial Culture Collection Covers Most Reactions Annotated to Human Microbiomes
Source: Microorganisms. 2020 Jun 27;8(7):966. doi: 10.3390/microorganisms8070966 (PMC7409220; doi:10.3390/microorganisms8070966)
Supplement: Supplementary file 1 [file microorganisms-08-00966-s001.zip › Supplementary_Table_S1.docx]

**Table S1.** Assembly and annotation statistics for the Liebefeld selection strains. They are published as BioProject PRJNA543085.

| **Strain** | **Species** | **BioSample** | **GenBank** | **Library preparation protocol** | **Sequencing technology I** | **Sequencing technology II** | **No. of reads** | **Sequencing Coverage (approx.)** | **Genome size [bp]** | **Largest scaffold [bp]** | **No. of scaffolds** | **n50 [bp]** | **No. of genes** | **BUSCO complete single copy orthologs** |
| --- | --- | --- | --- | --- | --- | --- | --- | --- | --- | --- | --- | --- | --- | --- |
| FAM19036 | *Acidipropionibacterium acidipropionici* ^1,4^ | SAMN11653933 | CP040634 | PacBio DNA Template Prep Kit 2.0 | PacBio RSII | RSII P4/C2 chemistry | 123’170 | 119 x | 3’622’795 | 3’622’795 | 1 | 3’622’795 | 3’316 | 98.9% |
| FAM19038 | *Acidipropionibacterium jensenii* ^1,4^ | SAMN11653934 | CP040635 | PacBio DNA Template Prep Kit 2.0 | PacBio RSII | RSII P4/C2 chemistry | 106’592 | 117 x | 2’999’408 | 2’999’408 | 1 | 2’999’408 | 2’645 | 98.9% |
| FAM24227 | *Facklamia tabacinasalis* ^3,4^ | SAMN11653935 | VBSP00000000 | TruSeq DNA PCR-free | Illumina (150 x 150) | HiSeq3000 | 4’103’050 | 489 x | 2’518’736 | 195’929 | 1 | 49’211 | 2’366 | 91.4% |
| FAM20446 | *Lactobacillus casei* ^1^ | SAMN11653936 | VBSQ00000000 | TruSeq DNA PCR-free | Illumina (150 x 150) | HiSeq3000 | 5’089’275 | 518 x | 2’946’858 | 141’905 | 147 | 59’012 | 2’899 | 98.6% |
| FAM21277 | *Lactobacillus delbrueckii ssp. bulgaricus* ^1^ | SAMN11653937 | VBSR00000000 | TruSeq DNA PCR-free | Illumina (150 x 150) | HiSeq3000 | 218’653 | 32 x | 2’019’838 | 91’667 | 199 | 24’499 | 1’919 | 97.5% |
| FAM21784 | *Lactobacillus delbrueckii ssp. lactis* ^1^ | SAMN11653938 | VBSS00000000 | TruSeq DNA PCR-free | Illumina (150 x 150) | HiSeq3000 | 194’460 | 29 x | 2’005’415 | 121’218 | 130 | 33’472 | 1’915 | 97.7% |
| FAM19471 | *Lactobacillus fermentum* ^1^ | SAMN11653939 | VBST00000000 | TruSeq DNA PCR-free | Illumina (150 x 150) | HiSeq3000 | 4’100’565 | 604 x | 2’036’472 | 154’742 | 211 | 34’842 | 1’964 | 96.6% |
| FAM22472 | *Lactobacillus helveticus* ^1^ | SAMN11653940 | VBSU00000000 | TruSeq DNA PCR-free | Illumina (150 x 150) | HiSeq3000 | 1’253’869 | 187 x | 2’006’476 | 106’038 | 95 | 44’385 | 2’124 | 99.1% |
| FAM23169 | *Lactobacillus parabuchneri* ^1^ | SAMN11653941 | VBSV00000000 | TruSeq DNA PCR-free | Illumina (150 x 150) | HiSeq3000 | 2’653’967 | 284 x | 2’800’230 | 351’075 | 133 | 76’021 | 2’634 | 98.0% |
| FAM3257 | *Lactobacillus paracasei* ^1^ | SAMN11653942 | VBSW00000000 | TruSeq DNA | Illumina (100 x 100) | HiSeq2000 | 10’120’781 | 646 x | 3’133’908 | 692’651 | 54 | 331’758 | 3’078 | 99.1% |
| FAM1079 | *Lactobacillus parafarraginis* ^1^ | SAMN11653943 | VBSX00000000 | TruSeq DNA PCR-free | Illumina (150 x 150) | HiSeq3000 | 2’802’019 | 284 x | 2’963’205 | 171’272 | 89 | 68’638 | 2’810 | 98.4% |
| FAM21789 | *Lactobacillus plantarum* ^1^ | SAMN11653944 | VBSY00000000 | TruSeq DNA PCR-free | Illumina (170 x 130) | HiSeq3000 | 3’348’986 | 309 x | 3’254’561 | 180’847 | 100 | 79’844 | 3’143 | 98.2% |
| FAM20558 | *Lactobacillus rhamnosus* ^1^ | SAMN11653945 | VBSZ00000000 | TruSeq DNA PCR-free | Illumina (150 x 150) | HiSeq3000 | 1’937’369 | 191 x | 3’040’665 | 407’633 | 49 | 250’015 | 2’899 | 98.6% |
| FAM17891 | *Lactococcus lactis ssp. cremoris* ^1^ | SAMN11653946 | VBTA00000000 | TruSeq DNA PCR-free | Illumina (150 x 150) | HiSeq3000 | 1’380’372 | 152 x | 2’718’424 | 488’026 | 68 | 180’997 | 2’707 | 98.6% |
| FAM17927 | *Lactococcus lactis ssp. lactis* ^1^ | SAMN11653947 | VBTB00000000 | TruSeq DNA PCR-free | Illumina (150 x 150) | HiSeq3000 | 5’801’548 | 631 x | 2’757’319 | 391’390 | 49 | 234’031 | 2’792 | 98.6% |
| FAM23217 | *Lactococcus raffinolactis* ^1^ | SAMN11653948 | VBTC00000000 | TruSeq DNA PCR-free | Illumina (150 x 150) | HiSeq3000 | 3’060’873 | 391 x | 2’347’098 | 227’418 | 74 | 105’993 | 2’289 | 97.5% |
| FAM18356 | *Leuconostoc mesenteroides* ^1^ | SAMN11653949 | VBTD00000000 | TruSeq DNA PCR-free | Illumina (150 x 150) | HiSeq3000 | 13’052’302 | 2092 x | 1’871’533 | 383’178 | 40 | 96’446 | 1’939 | 93.9% |
| FAM24235 | *Marinilactibacillus psychrotolerans* | SAMN11653950 | VBTE00000000 | TruSeq DNA PCR-free | Illumina (150 x 150) | HiSeq3000 | 3’009’679 | 320 x | 2’821’130 | 163’209 | 123 | 54’776 | 2’763 | 94.1% |
| FAM18969 | *Pediococcus acidilactici* ^1^ | SAMN11653951 | VBTF00000000 | TruSeq DNA PCR-free | Illumina (170 x 130) | HiSeq3000 | 3’170’828 | 458 x | 2’075’235 | 502’975 | 18 | 413’925 | 2’012 | 97.7% |
| FAM19144 | *Pediococcus pentosaceus* ^1^ | SAMN11653952 | VBTG00000000 | TruSeq DNA | Illumina (100 x 100) | HiSeq2000 | 12’892’178 | 1329 x | 1’940’593 | 424’819 | 26 | 325’547 | 1’940 | 98.0% |
| FAM18815 | *Pediococcus stilesii* ^2^ | SAMN11653953 | VBTH00000000 | TruSeq DNA | Illumina (100 x 100) | HiSeq2000 | 8’321’960 | 793 x | 2’097’950 | 257’971 | 57 | 120’403 | 2’080 | 97.3% |
| FAM14217 | *Propionibacterium freudenreichii* ^1^ | SAMN14943796 | CP053853 | PacBio DNA Template Prep Kit 2.0 | PacBio RSII | RSII P4/C2 chemistry | 128'043 | 169 x | 2'633'557 | 2'633'557 | 1 | 2'633'557 | 2'356 | 96.9% |
| FAM20833 | *Staphylococcus xylosus* ^1^ | SAMN11653955 | VBTJ00000000 | TruSeq DNA PCR-free | Illumina (150 x 150) | HiSeq3000 | 3’039’168 | 325 x | 2’805’510 | 1’404’038 | 13 | 1’404’038 | 2’613 | 99.6% |
| FAM13496 | *Streptococcus salivarius ssp. thermophilus* ^1^ | SAMN11653956 | VBTK00000000 | TruSeq DNA PCR-free | Illumina (150 x 150) | HiSeq3000 | 2’721’351 | 445 x | 1’836’358 | 136’567 | 62 | 57’048 | 1’945 | 98.6% |

^1^Species reported in human gastrointestinal microbiomes [61]. ^2^Species reported in human feces [62].  ^3^Species reported in the gut of rodents [63]. ^4^Not a lactic acid bacterium.
