# Supplementary material for: In Silico Comparison Shows that the Pan-Genome of a Dairy-Related Bacterial Culture Collection Covers Most Reactions Annotated to Human Microbiomes
Source: Microorganisms. 2020 Jun 27;8(7):966. doi: 10.3390/microorganisms8070966 (PMC7409220; doi:10.3390/microorganisms8070966)
Supplement: Supplementary file 1 [file microorganisms-08-00966-s001.zip › Supplementary_Table_S2.docx]

**Table S2.** Information about the individuals whose metagenomes were analyzed.

| **Sample Name** | **Country** | **Gender** | **Age** | **BMI** | **IBD** |
| --- | --- | --- | --- | --- | --- |
| MH0001 | Denmark | female | 49 | 25.55 | no |
| MH0002 | Denmark | female | 59 | 27.28 | no |
| MH0003 | Denmark | male | 69 | 33.19 | no |
| MH0004 | Denmark | male | 59 | 31.18 | no |

Based on Qin, 2010 [3]. Supplementary information: Table 1: DNA sample information.
