# Supplementary material for: In Silico Comparison Shows that the Pan-Genome of a Dairy-Related Bacterial Culture Collection Covers Most Reactions Annotated to Human Microbiomes
Source: Microorganisms. 2020 Jun 27;8(7):966. doi: 10.3390/microorganisms8070966 (PMC7409220; doi:10.3390/microorganisms8070966)
Supplement: Supplementary file 1 [file microorganisms-08-00966-s001.zip › Supplementary_Table_S3.docx]

**Table S3.** Information about sequencing data of the metagenomes.

| **Sample ID** | **No. of contigs** | **Contig N50 (bp)** | **Total length (Mb)** | **% reads assembled** | **Unassembled reads (Gb)** | **No. of genes ^1^** |
| --- | --- | --- | --- | --- | --- | --- |
| MH0001 | 14,301 | 1,618 | 19.69 | 46.34 | 1.06 | 28229 |
| MH0002 | 65,392 | 1,680 | 88.77 | 45.31 | 1.91 | 132423 |
| MH0003 | 68,658 | 2,640 | 119.59 | 54.4 | 1.72 | 157036 |
| MH0004 | 23,793 | 1,681 | 31.92 | 41.54 | 1.05 | 44743 |

Based on Qin, 2010 [3]. Supplementary information: Table 4, Summary of de novo assembly results.

^1^ The number of genes was calculated based on the annotation of the assembly described in section 2.2.
