# Supplementary material for: In Silico Comparison Shows that the Pan-Genome of a Dairy-Related Bacterial Culture Collection Covers Most Reactions Annotated to Human Microbiomes
Source: Microorganisms. 2020 Jun 27;8(7):966. doi: 10.3390/microorganisms8070966 (PMC7409220; doi:10.3390/microorganisms8070966)
Supplement: Supplementary file 1 [file microorganisms-08-00966-s001.zip › Supplementary_Table_S4.docx]

**Table S4.** Assembly and annotation statistics for the genomes of 24 human gut bacteria randomly selected from Zou et al. [41] (BioProject Accession: PRJNA482748).

| **Strain** | **BioSample** | **GenBank** | **Genome size [bp]** | **Largest scaffold [bp]** | **No. of scaffolds** | **n50 [bp]** | **No. of genes** |
| --- | --- | --- | --- | --- | --- | --- | --- |
| *Bacteroides sp. AM41-16* | SAMN09736530 | GCF_003603495.1 | 4’905’507 | 546’888 | 57 | 175’313 | 4’186 |
| *Bacteroides sp. AM56-10ce* | SAMN09736645 | GCF_003435255.1 | 7’053’306 | 298’410 | 298 | 66’442 | 6’142 |
| *Bacteroides stercoris* | SAMN09736614 | GCF_003464035.1 | 3’892’011 | 235’099 | 102 | 90’924 | 3’880 |
| *Bacteroides thetaiotaomicron* | SAMN09736625 | GCF_003463855.1 | 5’910’608 | 240’326 | 165 | 90’312 | 5’099 |
| *Bacteroides uniformis* | SAMN09736397 | GCF_003469025.1 | 4’874’190 | 942’010 | 51 | 227’321 | 4’140 |
| *Bacteroides vulgatus* | SAMN09736590 | GCF_003467095.1 | 4’884’071 | 301’669 | 131 | 84’060 | 4’428 |
| *Bacteroides xylanisolvens* | SAMN09734689 | GCF_003474245.1 | 6’027’336 | 325’539 | 98 | 166’063 | 8’606 |
| *Bifidobacterium adolescentis* | SAMN09734811 | GCF_003472095.1 | 2’193’162 | 832’581 | 12 | 485’133 | 1’765 |
| *Bifidobacterium bifidum* | SAMN09734857 | GCF_003471595.1 | 2’202’931 | 560’724 | 21 | 350’700 | 1’785 |
| *Bifidobacterium longum* | SAMN09736971 | GCF_003436505.1 | 2’414’802 | 356’420 | 51 | 134’171 | 2’026 |
| *Bifidobacterium pseudocatenulatum* | SAMN09736909 | GCF_003437075.1 | 2’383’735 | 657’836 | 17 | 232’905 | 1’987 |
| *Clostridium sp. AF22-10* | SAMN09734438 | GCF_003478725.1 | 3’344’693 | 321’559 | 86 | 155’136 | 3’392 |
| *Clostridium sp. AF34-10BH* | SAMN09734608 | GCF_003478095.1 | 4’001’893 | 422’643 | 84 | 106’489 | 3’291 |
| *Collinsella sp. TF11-5AC* | SAMN09736950 | GCF_003436775.1 | 2’329’201 | 263’055 | 50 | 96’513 | 1’976 |
| *Coprobacillus cateniformis* | SAMN09736764 | GCF_003439335.1 | 3’750’326 | 359’646 | 85 | 91’288 | 3’735 |
| *Dorea formicigenerans* | SAMN09736494 | GCF_003467665.1 | 3’367’825 | 741’683 | 44 | 213’283 | 3’203 |
| *Faecalibacterium sp. OF03-6AC* | SAMN09736700 | GCF_003478175.1 | 2’830’308 | 445’388 | 31 | 234’049 | 2’496 |
| *Prevotella copri* | SAMN09734839 | GCF_003471795.1 | 3’862’109 | 246’962 | 150 | 65’145 | 3’353 |
| *Propionibacterium sp. KPL2009* | SAMN09736719 | GCF_003482085.1 | 2’532’082 | 874’393 | 10 | 687’614 | 2’359 |
| *Roseburia intestinalis* | SAMN09736561 | GCF_003467035.1 | 4’337’927 | 364’418 | 91 | 146’443 | 3’463 |
| *Roseburia sp. AF12-17LB* | SAMN09734215 | GCF_003479605.1 | 2’971’866 | 589’952 | 35 | 254’992 | 2’431 |
| *Ruminococcus sp. AM12-48* | SAMN09734788 | GCF_003473905.1 | 4’148’882 | 347’846 | 87 | 136’039 | 3’247 |
| *Ruminococcus sp. AM36-17* | SAMN09736476 | GCF_003480205.1 | 4’459’680 | 295’337 | 86 | 119’606 | 3’489 |
| *Ruminococcus sp. AM54-14NS* | SAMN09736633 | GCF_003478265.1 | 4’060’909 | 244’553 | 131 | 86’405 | 3’451 |
